# The Case for Parent-Implemented Programs to Mitigate Musculoskeletal Complications in Children With Severe Cerebral Palsy in Resource-Limited Settings

**DOI:** 10.9745/GHSP-D-23-00463

**Published:** 2024-12-20

**Authors:** Shayne R. van Aswegen, Mark T. Richards, Brenda M. Morrow

**Affiliations:** a Department of Paediatrics and Child Health, University of Cape Town, Cape Town, South Africa.

## Abstract

Children with severe cerebral palsy are vulnerable to orthopedic complications, particularly in resource-limited settings, which can compound disability. A focus on home-based programs may help to improve their quality of life and participation.

## Key Messages

- Children with cerebral palsy in resource-limited settings are at increased risk of developing musculoskeletal deformities that diminish their quality of life.
- Program managers should equip caregivers to implement basic prevention strategies to help ensure social participation and inclusion.
- Policymakers should prioritize scalable and sustainable home-based training and the development of locally produced, low-cost technologies for program implementation.

## INTRODUCTION

Cerebral palsy (CP) is a leading cause of childhood motor disability worldwide. Secondary musculoskeletal (MSK) complications of CP are common and can further incapacitate children functionally and restrict their participation in ordinary life. Conventional, comprehensive management approaches for preventing complications of CP require regular access to specialized and coordinated medical interventions, rehabilitation, and equipment, all of which may be scarce or unavailable in resource-limited settings (RLSs).^1^ In this article, we present the case for a standardized home-based program aimed at preventing secondary MSK complications in children with severe CP living in RLSs.

**FIGURE 1:**
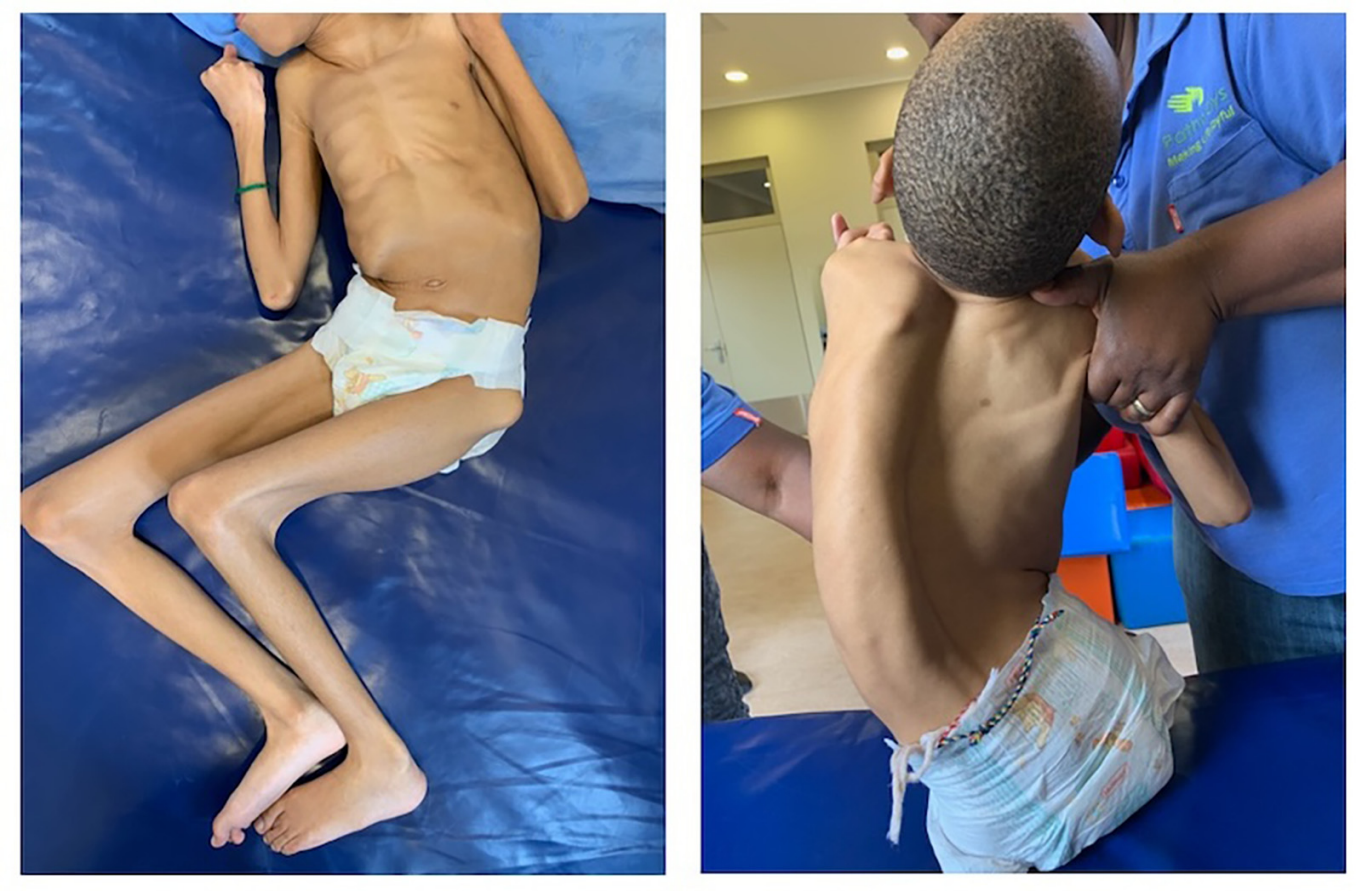
Common Musculoskeletal Complications of Severe Cerebral Palsy, as Seen in a Child Aged 11 Years With Severe Cerebral Palsy, Pathways Stimulation Centre, Durban, South Africa. © 2020 Shayne R. van Aswegen

The current birth prevalence of CP in low- and middle-income countries (LMICs) is estimated to be as high as 3.4 per 1000 births, compared to 1.6 per 1000 live births in high-income countries (HICs).^2^ Due to differences in maternal and perinatal health and the particular risk factors for CP in LMICs, these countries also tend to have higher proportions of children with more severe subtypes of CP,^3^ including bilateral spastic and dyskinetic presentations^4^^–^^7^ (i.e., more children functioning at the non- or partially ambulant Gross Motor Function Classification System levels [GMFCS III to V]).^8^ Although their MSK systems usually appear to be radiologically normal at birth, children with such severe neurological impairments are at increased risk of developing MSK complications, including hip displacement, muscle contractures, and scoliosis,^9^ which often present before the age of 5 years.^10^ Figure 1 shows the extent of MSK complications in a child aged 11 years visiting an outpatient clinic in an RLS, a presentation that is commonly seen.

### The Functional Impact of Secondary Musculoskeletal Complications in Cerebral Palsy

**FIGURE 2:**
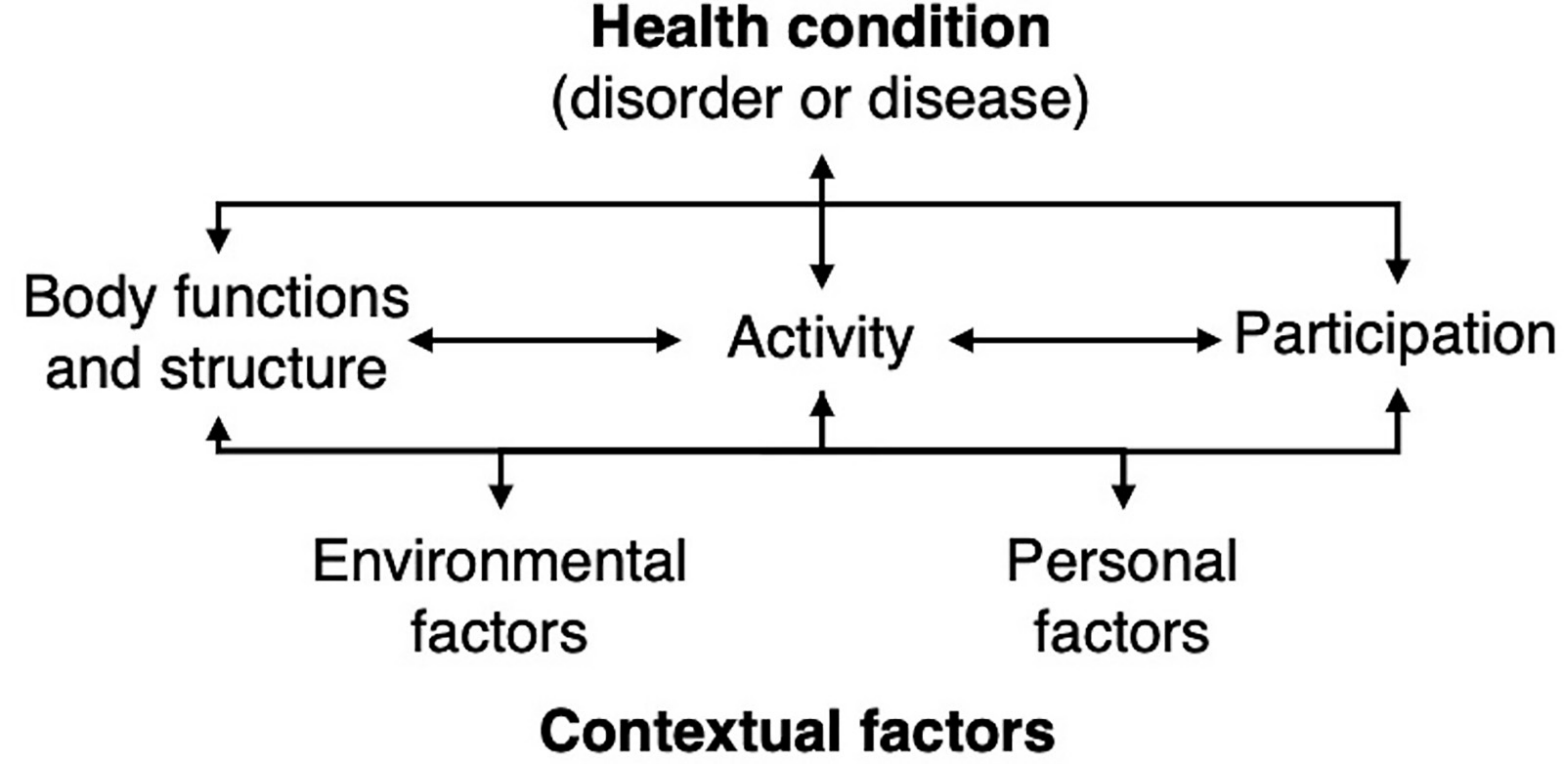
International Classification of Functioning, Disability and Health Framework Source: World Health Organization, 2002.^11^

The many ways that MSK complications affect the quality of life of the child and their family are best considered through the holistic lens of the International Classification of Functioning, Disability and Health framework (ICF)^11^ (Figure 2). Multiple intrinsic and extrinsic factors or domains converge to determine the overall functional status of an individual, notwithstanding the primary diagnosis or disorder, in this case, CP.

For example, as shown in the child in Figure 1, the child has developed secondary MSK deformities (ICF domain of body functions and structure) that profoundly restrict opportunities for age-appropriate activity and participation. Chronic, nociceptive, MSK pain, thought to be at least partially due to joint contractures, has been reported in more than 50% of children with severe spastic and dyskinetic CP.^12^ Pain and joint range limitations prevent the child from assuming sitting and standing positions in which most functional daily activities occur (ICF domain of activity) (e.g., sharing a family meal around a dining table), even where appropriate assistive devices are available. Therefore, the child will likely default to the lying position for extended periods, which, in turn, predisposes to further spinal and joint asymmetry.^13^ Daily activities, such as bathing, dressing, and cleaning the perineal area, become challenging for the caregiver and painful and distressing for the child. Safe feeding and drinking can be particularly difficult and may be a prolonged exercise due to the child’s oro-motor impairment and poor head control. Participation in other social spheres (e.g., enrollment in an educational institution) is likely very limited (ICF domain of participation) because the child cannot sit either for transportation or for the duration of the school day. Therefore, an increased focus on early prevention of MSK complications in this population is mandatory to provide activity and participatory opportunities for these children and their families.

## THE CHALLENGES OF CEREBRAL PALSY MANAGEMENT IN RESOURCE-LIMITED SETTINGS

### The Inadequacy of Current Guidelines

Current practice guidelines for children with CP all originate in HICs, where the disease burden of CP differs from LMICs. Thus, the guidelines neither focus on the severe presentations of CP nor make provision for an RLS, mainly due to a lack of research from these settings. The recommendations they do make presuppose access to regular therapy oversight, pharmacological and surgical management, and the availability of assistive technologies.^14^^,^^15^ Additionally, best therapy guidelines strongly promote early, child-active, task-oriented interventions, according to motor learning theory.^16^ If a child with CP has already developed serious MSK complications, opportunities for child-active functional gains are limited, even in places where there is access to rehabilitation services. Although surgical correction may be considered in some instances and where funds allow, it is often unfeasible, owing to the scale of remediation required, the poor general health of the child, and lack of access to adequate post-operative support.

Current practice guidelines for children with CP all originate in HICs and neither focus on the severe presentations of CP nor make provision for an RLS.

### The Contextual Challenges in a Resource-Limited Setting

Minimizing MSK complications relies on a timely, continuous, integrated approach, which is traditionally coordinated by a team of rehabilitation therapists and orthotists, a scarce commodity in RLSs. For example, in South Africa, a middle-income country with extremely high levels of inequality (Gini Coefficient of >60 in 2024^17^), up to one-third of therapists working in poorly-resourced, rural settings are brand new graduates performing their obligatory “community service” year.^18^ In addition to the multiple cultural and social barriers they often face, therapists are unlikely to have the training, experience, and tools to manage children with complex long-term health conditions in an RLS, where they are required to “do more with less.”^19^

In South Africa, CP surveillance is not yet standardized and is frequently either inconsistent or absent in peri-urban and rural areas. We have come across many children in these settings that are simply missed or lost to follow-up. In these cases, the onus is on caregivers to bring their children to health facilities for rehabilitation and checkups, which requires both a mode of appropriate and affordable transport and a level of health awareness and literacy most would not possess.^20^ Caregivers frequently report that they struggle to cope with the care of their child with CP, and the perceived burden of care often relates to difficulties with activities of daily living (e.g., feeding), lack of knowledge about the condition, and general feelings of inadequacy.^21^^,^^22^ In RLSs, the inexperience and knowledge gaps of primary health workers in identifying the families in need and not feeling equipped to support them compound these issues.^23^

## IMPROVING CARE FOR CHILDREN WITH CEREBRAL PALSY IN RESOURCE-LIMITED SETTINGS

### The Benefits of Caregiver-Implemented Programs

Instead of trying to increase coverage of formal rehabilitative health services in these areas, we propose that existing health delivery structures in RLSs be strengthened to deliver a standardized, community-led program that is built around the prevention of MSK sequelae in CP. Recruiting primary caregivers to perform simple, cost-effective interventions in the child’s natural environment is pragmatic because it reduces access barriers (e.g., transport costs) and reliance on the availability of specialized rehabilitative services. Importantly, it is also in line with the concept of family-centered care.^24^ Supported by trained community health workers (CHWs) already based in RLSs who are, in turn, supported by rehabilitation therapists, caregivers would be empowered to take an active role in decision-making, setting functional goals, and delivering effective interventions. There is already an increasing recognition of the value of home-based intervention programs (HBIPs), even in HICs, where primary caregivers are coached by health professionals to implement essential parts of the therapy program.^25^^,^^26^ Community-based initiatives usually require the establishment of local support networks for implementation and/or sustainability. These groups would benefit the families involved and help to increase knowledge of the condition and address cultural barriers, such as stigma, which are significant in African RLSs.^27^ This would increase the likelihood of acceptability and program adherence.

We propose that existing health delivery structures in RLSs be strengthened to deliver a standardized, community-led program that is built around the prevention of MSK sequelae in CP.

An example of a successful intervention that demonstrates this type of model is the study undertaken by Zuurmond et al. on the impact of an 11-month community-based caregiver training program on the quality of life of caregivers living in an RLS in Ghana.^28^ The study found significant improvements in caregiver knowledge and confidence in caring for their child, which included feeding practices, and caregivers reported that their children exhibited improvements in their physical and psychological health.

### Preventing Musculoskeletal Complications in Cerebral Palsy

The development of an HBIP should be informed by existing evidence of effective interventions that prevent MSK complications in CP and feasibility for a low-resource setting must be considered. A few comprehensive clinical guidelines exist that specifically focus on preventing MSK complications,^29^^–^^31^ and a few systematic reviews of therapeutic interventions aimed to guide clinical practice.^14^^,^^15^^,^^32^ The following nonsurgical, non-pharmacological, and low-tech physical modalities are included in these guidelines and reviews.

- 24-hour postural management, including lying, adapted seating, and supported standing using appropriate assistive devices.^14^^,^^15^^,^^29^^–^^32^
- Orthotics or splints for upper and lower limbs to prevent loss of joint range of motion and to assist with standing and walking (lower limbs).^14^^,^^15^^,^^29^^–^^32^
- Low-load muscle stretching.^15^^,^^29^^,^^30^^,^^32^

These interventions ideally require continuity and integration into everyday functional routines.^33^^–^^35^ Caregivers are required to move, feed, bathe, position, and dress a child with severe impairment many times a day, and we have observed them struggling to cope. Helping them modify their caregiving practices to promote MSK symmetry and flexibility using these simple techniques should reduce their burden of care and offer greater participation opportunities for the child.

A potential challenge would be the current cost and availability of the required equipment for postural management and splinting, which is significant for children in GMFCS level IV and V.^36^ Currently, in South Africa, local production of assistive technology is limited, and it is our experience that assistive devices are often imported, rendering them too costly for patients in the public sector.

### Lessons Learned From Existing Home-Based Initiatives for Cerebral Palsy in Resource-Limited Settings

Although no empirical studies have specifically evaluated the effectiveness of HBIPs in preventing MSK complications in severe CP, there is evidence that parent-implemented programs are acceptable and effective in improving other child and caregiver outcomes^37^ when accompanied by adequate professional training and support.^38^ Qualitative studies from RLSs suggest that, despite poverty and low caregiver education levels, parent-implemented intervention programs positively influence posture, positioning, self-care, feeding, and social function in children with severe CP. The intervention programs also significantly reduce parental stress because parents understand more about the condition and have increased confidence in their handling skills.^28^^,^^39^

From the caregivers’ perspectives, factors affecting their adherence to HBIPs include the presence of ongoing trusting and supportive relationships between the child, caregiver, and health care professionals and the ability to establish context-specific coping strategies.^38^^,^^40^ The program should have an element of fun, be able to be integrated into the family’s daily routines, and provide clear instructions and demonstrations for techniques and activities.^41^ Health care professionals value effective communication with families, regular joint goal-setting to maximize compliance, and specific training in clinical skills and using resources for CP management in an RLS.^42^

## POLICY RECOMMENDATIONS AND IMPLICATIONS

### Principles of Implementation

1. The success of an HBIP depends on developing a context-specific application; therefore, any initiative should be informed by an a priori analysis of the target population, including all stakeholders (e.g., caregivers, CHWs, and other health care professionals that work with families with children who have CP).
2. The program should be developed by a team of expert clinicians with appropriate experience in CP management as part of a standardized, comprehensive early intervention care package for this population.
3. An important prerequisite for the program would be the provision of affordable, appropriate assistive technologies. Thus, the development and/or local production of low-tech, low-cost assistive devices should be prioritized. Appropriate paper-based technology devices have been effective options in RLSs.^43^ Some devices may even be made or adapted from readily available household equipment, such as PVC piping and recycled material. Regardless of the materials used, equipment should be as simple as possible, durable, and easy to apply in the correct manner.
4. For effective prevention of deformities, the HBIP should be commenced early in the child’s life, even before an official diagnosis of CP is made because, in LMICs, this process can be delayed by several years. The local health service delivery platform for a particular setting will dictate how the program is initiated, supported, and maintained, but we recommend that infants at high risk of CP or with neurodevelopmental delay or a firm diagnosis of CP be added to an “at risk” register that is kept at the district level or wherever therapist trainers are located and that these caregivers receive the training as early as possible. Considering that the main beneficiaries of this HBIP are children with severe impairment, we would recommend a reevaluation of children at age 2 years when GMFCS level has stabilized;^45^ however, in most children, GMFCS will not change, especially in more severely affected infants.^46^

The success of an HBIP depends on developing a context-specific application.

### Recommendations for a Home-Based Intervention Program for Severe Cerebral Palsy in Resource-Limited Settings

#### The Elements of the Program

The daily program should include the following components.

- 24-hour postural management regimes using variations of lying, sitting, and standing positions according to current clinical guidelines. This should be commenced from infancy or when diagnosis of CP or risk is established. Recommended low-cost assistive devices would include a side-lyer or weighted positioning cushions for lying, a positioning chair for sitting activities, and a simple wooden stander.
- Stretching and splinting regimes according to current clinical guidelines. For resting (sustained) stretching of both lower and upper limbs, thermoplastic splints or fabric gaiters can be used, if available, or firm foam can be covered with breathable materials and wrapped around the joints. To maintain a child’s ankle alignment during standing weight-bearing, ankle boots may suffice for a young child. For older children, thermoplastic splints or a caliper may be required.
- Handling skills and positioning options for daily activities, such as feeding, bathing, and dressing. A plastic bathing chair and a feeding and/or activity chair should be provided to facilitate care and promote activity. The child would also need a wheelchair or posture support buggy for mobility and to allow social engagement.

#### The Training and Post-Training Support of the Program

- The training should be delivered by rehabilitation therapists who can demonstrate the basic techniques but who would be able to adapt techniques and application of basic equipment for individual children as needed.
- If therapists are not available on-site, and if sufficient mobile networks are in place, guided video instruction may be feasible via telehealth platforms.
- Along with practical demonstrations of techniques and equipment use, a theoretical module on CP as a condition, the life course, management options, and prognosis should be mandatory.
- Low-tech training and reference materials, such as printed booklets with predominantly graphic illustrations of techniques, would be preferable in these settings where literacy may be low. Artificial intelligence and other mobile applications may also be used for post-training support, especially if available in local languages.
- Training should occur in a small group setting to encourage the formation of caregiver support groups. A CHW based in the area should attend the training with the caregivers they will be supporting.

#### The Community Support Network

- A support network should include therapists, where available, and nursing staff who should provide home visits at intervals to ensure that caregivers are managing the program effectively and oversee the work of the CHWs who provide primary-level support for families. Zuurmond et al. found that monthly telephone or in-person check-ins with caregivers and a group chat via social media for program coordinators were effective support mechanisms for their community-based program in Ghana.^28^
- Community engagement with local religious bodies, nongovernmental organizations, educational institutions, and other community players should be facilitated by social workers and/or via established community processes, which may be culturally and socio-geographically variable.
- Measures of program adherence and process (e.g., CHW visits, evidence of intervention adherence, such as postural management), clinical outcome measures (e.g., range of joint motion, pain, and incidence of other MSK complications), and participation indicators (e.g., frequency of attendance at school and other social activities) should be collected by CHWs and evaluated by therapists using a quality improvement process that allows for continuous and responsive feedback, adaptation, and implementation processes as new evidence emerges.

## CONCLUSION

Policymakers and program managers in RLSs should prioritize home-based packages of care for children with severe CP in these settings to mitigate the MSK consequences of CP, relieve caregiver burden, and ultimately improve the quality of life of those with and affected by CP. We have demonstrated that building capacity at a community level is feasible by prioritizing training and the development of low-cost technologies. We believe this is achievable through key partnerships with international health entities and nongovernmental organizations. As stated in the Sustainable Development Goals, we have an ethical obligation to ensure that no one with health needs is left behind and “ensure healthy lives and promote well-being for all at all ages” in all sociogeographic contexts by 2030.^46^

## Notes

**Peer Reviewed**

**First Published Online:** November 11, 2024.
